# Aging amorphous/crystalline heterophase PdCu nanosheets for catalytic reactions

**DOI:** 10.1093/nsr/nwz078

**Published:** 2019-07-05

**Authors:** Hongfei Cheng, Nailiang Yang, Xiaozhi Liu, Qinbai Yun, Min Hao Goh, Bo Chen, Xiaoying Qi, Qipeng Lu, Xiaoping Chen, Wen Liu, Lin Gu, Hua Zhang

**Affiliations:** 1 Center for Programmable Materials, School of Materials Science and Engineering, Nanyang Technological University, Singapore 639798, Singapore; 2 State Key Laboratory of Biochemical Engineering, Institute of Process Engineering, Chinese Academy of Sciences, Beijing 100190, China; 3 Beijing National Laboratory for Condensed Matter Physics, Institute of Physics, Chinese Academy of Sciences, Beijing 100190, China; 4 School of Physical Sciences, University of Chinese Academy of Sciences, Beijing 100049, China; 5 Singapore Institute of Manufacturing Technology, A*STAR, Singapore 638075, Singapore; 6 School of Materials Science and Engineering, University of Science and Technology Beijing, Beijing 100083, China; 7 School of Chemical and Biomedical Engineering, Nanyang Technological University, Singapore 637459, Singapore; 8 Songshan Lake Materials Laboratory, Dongguan 523808, China; 9 Department of Chemistry, City University of Hong Kong, Hong Kong, China

**Keywords:** amorphous/crystalline heterophase, nanosheets, aging, selective hydrogenation, catalysis

## Abstract

Phase engineering is arising as an attractive strategy to tune the properties and functionalities of nanomaterials. In particular, amorphous/crystalline heterophase nanostructures have exhibited some intriguing properties. Herein, the one-pot wet-chemical synthesis of two types of amorphous/crystalline heterophase PdCu nanosheets is reported, in which one is amorphous phase-dominant and the other one is crystalline phase-dominant. Then the aging process of the synthesized PdCu nanosheets is studied, during which their crystallinity increases, accompanied by changes in some physicochemical properties. As a proof-of-concept application, their aging effect on catalytic hydrogenation of 4-nitrostyrene is investigated. As a result, the amorphous phase-dominant nanosheets initially show excellent chemoselectivity. After aging for 14 days, their catalytic activity is higher than that of crystalline phase-dominant nanosheets. This work demonstrates the intriguing properties of heterophase nanostructures, providing a new platform for future studies on the regulation of functionalities and applications of nanomaterials by phase engineering.

## INTRODUCTION

Tremendous efforts have been devoted to the preparation of ultrathin 2D nanomaterials by wet-chemical methods owing to their unique physicochemical properties and promising applications [1–6]. As a member of the 2D family, ultrathin 2D noble-metal-based nanomaterials [7,8] have exhibited some intriguing behavior and excellent performance in terms of electrical properties [9,10], optical properties [11–14], photothermal therapy [15,16], catalysis [17–21], and so on.

Phase engineering is emerging as a promising and challenging research field in noble-metal-based nanomaterials [22,23]. In particular, as one kind of heterophase structure, the crystal-phase heterostructure [1], consisting of two different crystal phases, has demonstrated some unique properties and potential catalytic applications [24,25]. Recently, as another kind of heterophase structure, amorphous/crystalline heterophase structures have been prepared, showing promising catalytic performance [26–28]. As a typical example, we have successfully synthesized a series of amorphous/crystalline heterophase Pd nanosheets by the wet-chemical method, exhibiting heterophase-dependent chemoselectivity and catalytic activity [26]. This work presents a novel synthetic method for monometallic heterophase catalysts. As known, it has been widely reported that bimetallic/multimetallic nanomaterials can exhibit enhanced catalytic performance compared to monometallic components, arising from the synergistic effects between different elements [20,29]. Herein, for the first time, we have prepared two types of amorphous/crystalline heterophase PdCu nanosheets, of which one is amorphous phase-dominant and the other one is crystalline phase-dominant. Since the amorphous phase in metals tends to transform into the crystalline phase under ambient conditions, the phase transformation behavior of our synthesized heterophase PdCu nanosheets and the heterophase-dependent properties have been systematically studied. As a proof-of-concept application, the heterophase PdCu nanosheets are used as catalysts for the hydrogenation of 4-nitrostyrene. As a result, the amorphous phase-dominant nanosheets initially show excellent chemoselectivity. After aging for 14 days, their catalytic activity exceeds the directly synthesized crystalline phase-dominant nanosheets.

## RESULTS AND DISCUSSION

**Figure 1.**
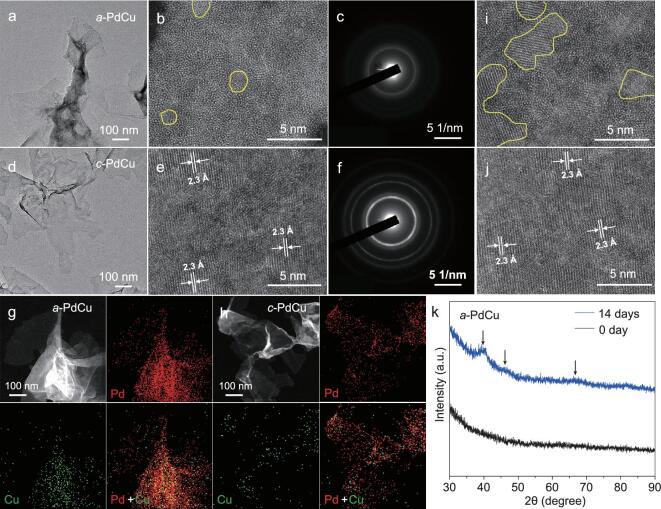
(a) TEM image, (b) *C*_S_-corrected STEM-HAADF image (the areas within yellow curves are crystalline domains), and (c) SAED pattern of the *a*-PdCu nanosheets. (d) TEM image, (e) *C*_S_-corrected STEM-HAADF image, and (f) SAED pattern of the *c*-PdCu nanosheets. (g, h) Dark-field STEM image and the corresponding EDX elemental mappings of the *a*-PdCu and *c*-PdCu nanosheets, respectively. (i, j) The *C*_S_-corrected STEM-HAADF images of *a*-PdCu and *c*-PdCu after aging for 14 days, respectively. The areas within yellow curves are crystalline domains. (k) XRD patterns of *a*-PdCu samples being aged for 0 day (i.e. the as-synthesized sample) and 14 days. The arrows indicate the weak peaks, confirming the slightly increased crystallinity of *a*-PdCu after aging for 14 days.

For the first time, amorphous/crystalline heterophase PdCu nanosheets with high purity (Fig. S1 in the online supporting information) have been synthesized (see the ‘Experimental’ section in the online supporting information for details). The amorphous phase-dominant PdCu nanosheets (denoted as *a*-PdCu, Figs 1a, S1a, b) were synthesized at a reaction temperature of 40°C. The spherical aberration-corrected scanning transmission electron microscopy high-angle annular dark-field (*C*_S_-corrected STEM-HAADF) image (Fig. 1b) shows that most of the area of the obtained nanosheet is amorphous, which is further proved by the weak diffuse ring in the selected area electron diffraction (SAED) pattern (Fig. 1c) and the absence of peaks in the X-ray diffraction (XRD) pattern (Fig. S2). When the reaction temperature changed to 80°C, the crystalline phase-dominant PdCu nanosheets (denoted as *c*-PdCu, Figs 1d, S1c, d) were synthesized. The *C*_S_-corrected STEM-HAADF image shows that most of the area of the obtained nanosheet is crystalline (Fig. 1e), and the average interplanar spacing of most crystalline areas is measured as 2.3 Å, which can be assigned to the 1/3(422) spacing of *fcc* structure but is slightly smaller than that of Pd [15], suggesting that the majority of the exposed facets are the {111} plane [15,20] and Cu atoms are incorporated into the Pd lattice. The good crystallinity is further proved by the clear diffraction rings in the SAED pattern (Fig. 1f) and the strong peaks in the XRD pattern (Fig. S2). Compared with pure Pd (PDF#00–046-1043), the XRD peaks of *c*-PdCu are slightly shifted to higher angles, indicating a smaller crystal lattice due to the incorporation of Cu atoms. Both types of PdCu nanosheets have an ultrathin thickness of ∼1.2 nm, as measured by the folded edges in TEM images (Fig. S3). This value is ∼2 nm thinner than that measured by atomic force microscopy (AFM) (Fig. S4), due to the presence of ligands on the surfaces of nanosheets [26,30]. In both *a*-PdCu and *c*-PdCu, the Pd and Cu atoms are uniformly distributed through the whole nanosheets (Fig. 1g, h), and the Cu content is ∼12 at% based on both energy-dispersive X-ray spectroscopy (EDX) (Fig. S5) and inductively coupled plasma optical emission spectroscopy (ICP-OES).

After aging, i.e. being stored in hexane under ambient conditions, for 14 days, the morphology of both *a*-PdCu and *c*-PdCu remained unchanged (Fig. S6), and there was no observable element segregation or leakage during the aging process (Fig. S7). However, more crystalline areas were observed in *a*-PdCu (Fig. 1i), while no obvious change was observed in *c*-PdCu and the exposed facets remained as {111} plane (Fig. 1j). In the XRD pattern of *a*-PdCu (Fig. 1k), some weak peaks appeared, further indicating that the crystallinity of *a*-PdCu slightly increased. Noble metals normally have crystalline structures due to strong metallic bonding [31]; hence their amorphous phase is thermodynamically unstable and tends to transform into a crystalline phase.

**Figure 2.**
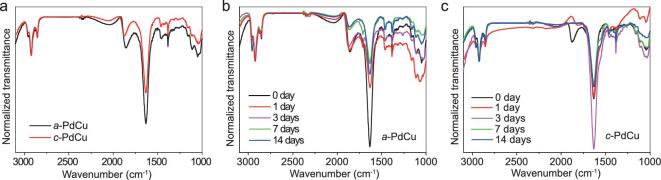
(a) FTIR spectra of the as-synthesized *a*-PdCu and *c*-PdCu. FTIR spectra of (b) *a*-PdCu and (c) *c*-PdCu with different aging times.

During the aging process, Fourier transform infrared spectroscopy (FTIR) was used to monitor the change of surface ligands on the heterophase PdCu nanosheets. The normalized FTIR spectra of *a*-PdCu and *c*-PdCu are shown in Fig. 2. For the as-synthesized *a*-PdCu and *c*-PdCu nanosheets (Fig. 2a), the peaks located within 2800–3000 cm^−1^ are attributed to the –CH_2_– stretching [32,33], mainly arising from the alkyl chain of octanoic acid (Fig. S8a). In the FTIR spectrum of Mo(CO)_6_ (Fig. S8b), there is a strong and broad absorption band centered at ∼1900 cm^−1^, which is assigned to the CO coordination groups [34]. Therefore, in the FTIR spectra of PdCu nanosheets, the broad band centered at ∼1860 cm^−1^ is assigned to the absorption of CO groups due to the decomposition of Mo(CO)_6_, indicating that the intermediate complexes containing CO groups are also present on the surface of the final products. This is consistent with nanomaterials synthesized using CO gas as the reductant, in which CO is present on the products [15,35]. For the as-synthesized samples (Fig. 2a), *a*-PdCu showed a relatively stronger absorption peak of CO groups as compared to the *c*-PdCu, implying that the amorphous phase-dominant nanosheets adsorbed more CO groups.

As the aging time increased, the relative intensity of CO absorption for both *a*-PdCu (Fig. 2b) and *c*-PdCu (Fig. 2c) gradually decreased with respect to the –CH_2_– absorption. However, after aging for 14 days, the CO absorption was still observed in the FTIR spectrum of *a*-PdCu, while it disappeared in the FTIR spectrum of *c*-PdCu. As discussed above, although the crystalline domains in *a*-PdCu increased after aging for 14 days, the crystallinity of *a*-PdCu is still lower than that of *c*-PdCu, resulting in the different ratios of the adsorbed CO to octanoic acid on their surfaces.

**Figure 3.**
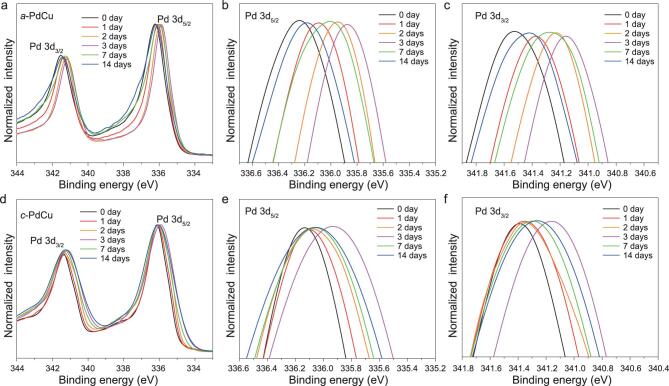
(a) XPS spectra of Pd 3d in *a*-PdCu samples with different aging times. (b, c) Magnification of normalized Pd 3d_5/2_ and Pd 3d_3/2_ peaks in (a), respectively. (d) XPS spectra of Pd 3d in *c*-PdCu samples with different aging times. (e, f) Magnification of normalized Pd 3d_5/2_ and Pd 3d_3/2_ peaks in (d), respectively.

The aging effects on the electronic properties of heterophase PdCu nanosheets were analyzed by X-ray photoelectron spectroscopy (XPS). The XPS results (Figs 3, S9) show that the Pd element in both *a*-PdCu and *c*-PdCu is predominantly in a metallic state. Comparing the as-synthesized samples, the Pd element in *a*-PdCu shows higher binding energy compared to *c*-PdCu (Fig. S9). Eventually, after aging for 14 days, although the binding energy peaks of *a*-PdCu shifted to lower values, they are still higher than that of *c*-PdCu (Fig. S9). This result is consistent with previous works [26,36] and could be due to the differences of crystallinities and surface ligands. As the aging time increased, both the *a*-PdCu and *c*-PdCu samples show similar trends in the binding energy change of Pd. During aging, in the first three days, the binding energy peaks shifted to lower values (red shift) (Fig. 3), due to not only the increase in crystallinity but also the desorption of CO groups, as the CO groups are electron withdrawing and can result in higher binding energy of the underlying metal atoms. From the third day onwards, the binding energy peaks shifted to higher values (blue shift) (Fig. 3), which could be due to the adsorption of oxygen [37]. During the aging process, the surface atoms of PdCu nanosheets were gradually oxidized, as shown by the deconvoluted XPS spectra of the *a*-PdCu and *c*-PdCu samples, in which the relative intensity of Pd^2+^/Pd^0^ clearly increased after aging for 14 days (Fig. S10). Surface oxidation can greatly increase the distance of nearest-neighbor Pd–Pd bonds and lead to a significant charge transfer from Pd to O [37], which could also cause the binding energy peaks of Pd to shift to higher energy levels. In fact, all these processes, including the crystallinity increase, desorption of ligands, and surface oxidation, took place concurrently during the aging process, but the dominant process may be different in the whole aging process; thus the binding energy peaks showed a red shift followed by a blue shift.

**Figure 4.**
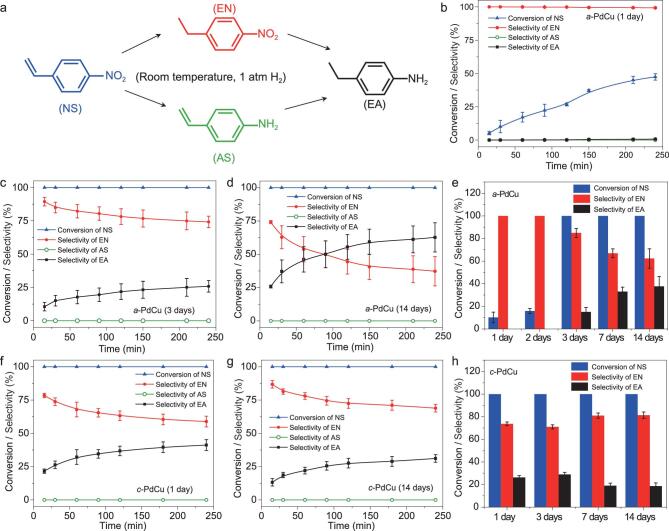
(a) Hydrogenation reaction of NS. (b–d) The kinetic curves showing the catalytic performance of *a*-PdCu after aging for 1 day, 3 days, and 14 days, respectively. (e) The catalytic results of 30 min hydrogenation reaction time using *a*-PdCu with different aging time as catalysts. (f, g) The kinetic curves showing the catalytic performance of *c*-PdCu after aging for 1 day and 14 days, respectively. (h) The catalytic results of 30 min hydrogenation reaction time using *c*-PdCu with different aging time as the catalysts.

As known, Pd and Pd-based alloys possess good catalytic performance in hydrogenation reactions [29,38,39], which are very important in the industrial and fine chemical fields [40,41]. Herein, as a proof-of-concept application, we studied the

aging effect on the selective hydrogenation of 4-nitrostyrene (NS), which is commonly used as a model reaction [42,43], using the heterophase PdCu nanosheets as catalysts. The hydrogenation reaction of NS was conducted at room temperature in one atm H_2_ atmosphere (see the ‘Experimental’ section in the online supporting information for details), and all the possible products are shown in Fig. 4a. 1-ethyl-4-nitrostyrene (EN) and 4-aminostyrene (AS) are the products of the hydrogenation of C=C and –NO_2_, respectively, and 4-ethylbenzenamine (EA) is the fully hydrogenated product. To understand the catalytic activity with respect to the aging time of heterophase PdCu nanosheets, the amount of catalyst was kept low so that the activity differences during the hydrogenation reaction were significant (Figs 4b-d, f, g, S11, S12). In addition, based on the kinetic curves of the catalytic hydrogenation, since the catalysts will lose their activity with extended reaction time (the conversion/selectivity curves tend to be flat after 120 min reaction time), the conversion of substrate and the selectivity of products within 30 min reaction time (Fig. 4e, h) were used as indicators of catalytic activity in the following discussion. After aging for one day, *a*-PdCu showed very high selectivity (>99.9%) towards EN, which was maintained even after 4 h reaction time (Fig. 4b). This high selectivity towards EN was maintained after aging for two days (Fig. S11a); meanwhile, the conversion rate of NS was increased, indicating that the catalytic activity was increased. After aging for three days and onwards, *a*-PdCu did not show selectivity towards EN, generating both EN and EA (Figs 4c, d, S11b), but the catalytic activity still gradually increased with time, as evidenced by the increased generation rate of the fully hydrogenated product, i.e. EA (Fig. 4e). In contrast, the *c*-PdCu nanosheets showed different behavior during the aging process and did not show chemoselectivity towards EN through the whole aging process (Figs 4f, g, S12). As for the catalytic activity, based on the selectivity of the fully hydrogenated product, i.e. EA, the catalytic activity first slightly increased after aging from one day to three days, and then it slowly decreased (Fig. 4h).

In the as-synthesized heterophase *a*-PdCu and *c*-PdCu nanosheets, the different crystallinities and different surface ligands result in different electronic structures of the surface metal atoms, which eventually lead to different selectivities in the catalytic hydrogenation reaction. This could also account for the selectivity change of the *a*-PdCu sample after aging for three days. Importantly, during the aging process, *a*-PdCu showed an increased catalytic activity and eventually surpassed the *c*-PdCu sample, which could result from their different amorphous/crystalline heterophase structures. In *a*-PdCu, although the crystallinity increased with prolonged aging time, the size of the crystalline domains is much smaller than that in the *c*-PdCu sample, as evidenced by the HRTEM image (Fig. 1i, j) and the weak peak in the XRD pattern (Fig. 1k). As a consequence, the amorphous/crystalline phase boundaries in the aged *a*-PdCu samples significantly increased compared with the as-synthesized *a*-PdCu, which could contribute to the enhanced catalytic activity [28].

## CONCLUSION

In conclusion, we have prepared two kinds of amorphous/crystalline heterophase PdCu nanosheets by a facile one-pot wet-chemical method, of which one is amorphous phase-dominant (*a*-PdCu) and the other one is crystalline phase-dominant (*c*-PdCu). Systematic studies were conducted to investigate the aging effect on the structures, properties and catalytic performance of the heterophase PdCu nanosheets. It was found that in the first two days of the aging process, *a*-PdCu showed very high chemoselectivity in the hydrogenation of 4-nitrostyrene, while *c*-PdCu did not show chemoselectivity. After three days of aging, *a*-PdCu lost the chemoselectivity, but its catalytic activity gradually increased, while the catalytic activity of *c*-PdCu gradually decreased and eventually became lower than that of *a*-PdCu after aging for 14 days. Based on the structural analysis and the FTIR and XPS results, the differences in chemoselectivity and catalytic activity of heterophase PdCu nanosheets could be attributed to the different amorphous/crystalline heterophase structures, surface ligands and binding energies. This work demonstrates the intimate correlation between heterophase structures and their physiochemical properties, which may inspire more discoveries in the preparation and mechanism studies of amorphous/crystalline heterophase structures. Importantly, the amorphous phase-dominant sample exhibited promising catalytic performance during the aging process, indicating that the metastable phase deserves intensive research efforts and that more interesting phase-dependent functions are expected to be discovered. This work on heterophase structures provides a strategy for phase engineering of nanomaterials for various promising applications.

## Supplementary Material

nwz078_Supplemental_File
