# Supplementary material for: Aging amorphous/crystalline heterophase PdCu nanosheets for catalytic reactions
Source: Natl Sci Rev. 2019 Jul 5;6(5):955–61. doi: 10.1093/nsr/nwz078 (PMC8291566; doi:10.1093/nsr/nwz078)
Supplement: nwz078_Supplemental_File [file nwz078_supplemental_file.docx]

Aging amorphous/crystalline heterophase PdCu nanosheets for catalytic reactions

Hongfei Cheng^1,†^, Nailiang Yang^1,2,†^, Xiaozhi Liu^3,4^, Qinbai Yun^1^, Min Hao Goh^5^, Bo Chen^1^, Xiaoying Qi^5^, Qipeng Lu^1,6^, Xiaoping Chen^7^, Wen Liu^7^, Lin Gu^3,4,8^, Hua Zhang^1,9,^*

^1^Center for Programmable Materials, School of Materials Science and Engineering, Nanyang Technological University, 50 Nanyang Avenue, Singapore 639798, Singapore

^2^State Key Laboratory of Biochemical Engineering, Institute of Process Engineering, Chinese Academy of Sciences, No. 1 Beiertiao, Zhongguancun, Beijing 100190, China

^3^Beijing National Laboratory for Condensed Matter Physics, Institute of Physics, Chinese Academy of Sciences, Beijing 100190, China

^4^School of Physical Sciences, University of Chinese Academy of Sciences, Beijing 100049, China

^5^Singapore Institute of Manufacturing Technology, A*STAR, 71 Nanyang Drive, Singapore 638075, Singapore

^6^School of Materials Science and Engineering, University of Science and Technology Beijing, Beijing 100083, China

^7^School of Chemical and Biomedical Engineering, Nanyang Technological University, 62 Nanyang Drive, Singapore 637459, Singapore

^8^Collaborative Innovation Center of Quantum Matter, Beijing 100190, China

^9^Department of Chemistry, City University of Hong Kong, Kowloon, Hong Kong, China

^†^These authors contributed equally to this work.

*Corresponding author. E-mail: hzhang@ntu.edu.sg; hua.zhang@cityu.edu.hk.

**Chemicals.**

Palladium(II) acetylacetonate (Pd(acac)_2_), copper(II) acetylacetonate (Cu(acac)_2_), molybdenum hexacarbonyl (Mo(CO)_6_), trioctylphosphine oxide (TOPO), and octanoic acid (≥ 98%) were purchased from Sigma-Aldrich. *N, N*-dimethylformamide (DMF) was purchased from Fisher Scientific. Hexane (95%) was purchased from Aik Moh. Ethanol (99.9%) was purchased from Merck. 4-nitrostyrene (NS) (stabilized by 4-tert-Butylcatechol) was purchased from Tokyo Chemical Industry Co., Ltd. All the chemicals were used as received without further purification.

**Synthesis of heterophase PdCu nanosheets.**

In order to synthesize the amorphous phase-dominant PdCu (*a*-PdCu) hetero-phase nanosheets, 20 mg of Pd(acac)_2_, 8 mg of Cu(acac)_2_, 260 mg of TOPO, and 16 mL of octanoic acid were added into a 50 mL round-bottom flask with magnetic stirring under N_2_ bubbling for 60 min. After 60 mg of Mo(CO)_6_ were added into the aforementioned mixture, it was magnetically stirred for 60 min. Then 3 mL of DMF were added and the mixture was stirred for 40 min under N_2_ atmosphere. After that, the flask was immersed into a water bath at 40 ^o^C and kept at 40 ^o^C for 20 min. After the flask was naturally cooled down to room temperature, hexane was added and the final product was obtained by centrifugation at 6,000 rpm for 5 min. The as-obtained nanosheets were washed twice with hexane, and then dispersed in hexane.

In order to synthesize crystalline phase-dominant PdCu (*c*-PdCu) nanosheets, 20 mg of Pd(acac)_2_, 2 mg of Cu(acac)_2_, 260 mg of TOPO, and 16 mL of octanoic acid were added into a 50 mL round-bottom flask with magnetic stirring under N_2_ bubbling for 60 min. After 20 mg of Mo(CO)_6_ and 3 mL of DMF were added into the aforementioned mixture, it was magnetically stirred for 60 min. Then the flask was immersed into a water bath at 80 ^o^C and kept at 80 ^o^C for 30 min. After the flask was naturally cooled down to room temperature, hexane was added and the final product was obtained by centrifugation at 6,000 rpm for 5 min. The as-obtained nanosheets were washed twice with hexane, and then dispersed in hexane.

**Aging experiment.**

The samples were stored in hexane at room temperature (25 °C). After aging for 1 day, 2 days, 3 days, 7 days and 14 days, a proper amount of sample was taken from the vial and used for characterizations.

**Catalytic hydrogenation reaction.**

In a typical experiment, 100 µL of PdCu catalyst in hexane (0.3 mg/mL) and 74.5 mg of NS were added into 2.5 mL of ethanol in a 5 mL flask. A H_2_ balloon was introduced after the flask was purged with H_2_ for 5 min. The reaction was performed at room temperature (25 °C). Liquid samples were analyzed by gas chromatography (GC) equipped with a flame ionization detector and an HP-CP8713 capillary column.

**Characterization.**

Transmission electron microscopy (TEM), high-resolution TEM (HRTEM), and dark-field scanning TEM (DF-STEM) images were taken on a JEOL JEM-2100F microscope. Selected area electron diffraction (SAED) patterns were recorded by JEOL JEM-2010 HR microscope. The spherical aberration-corrected STEM-high-angle annular dark-field (C_S_-corrected STEM-HAADF) images were taken from a JEOL ARM200F (JEOL, Tokyo, Japan) microscope equipped with a CEOS (Heidelberg, Germany) probe aberration corrector, operated at 200 keV. X-ray diffraction (XRD) patterns were recorded with a Shimadazu XRD-6000, using CuKα radiation (λ=1.5406 Å). The XRD samples were prepared by dropping the PdCu nanosheet suspension onto a glass substrate. The Fourier-transform infrared spectroscopy (FTIR) spectra of the PdCu nanosheets were collected using a FTIR spectrometer (Frontier, PerkinElmer). The FTIR samples were prepared by mixing the PdCu nanosheets with KBr powder and pressing the mixture into a pellet. The electronic binding energy of samples was examined by X-ray photoelectron spectroscopy (XPS) on a VG ESCALAB 220i-XL instrument with a monochromatic Al Kα (1486.7 eV) X-ray source. Binding energies were calibrated by setting the binding energy of C 1s to 284.8 eV. The XPS samples were prepared by dropping the PdCu nanosheet suspension onto a silicon substrate. The concentration of Pd was measured by the inductively coupled plasma optical emission spectrometry (ICP-OES, PerkinElmer, Optima 8000). The reaction was tracked by GC (Agilent GC-FID 5890).

**
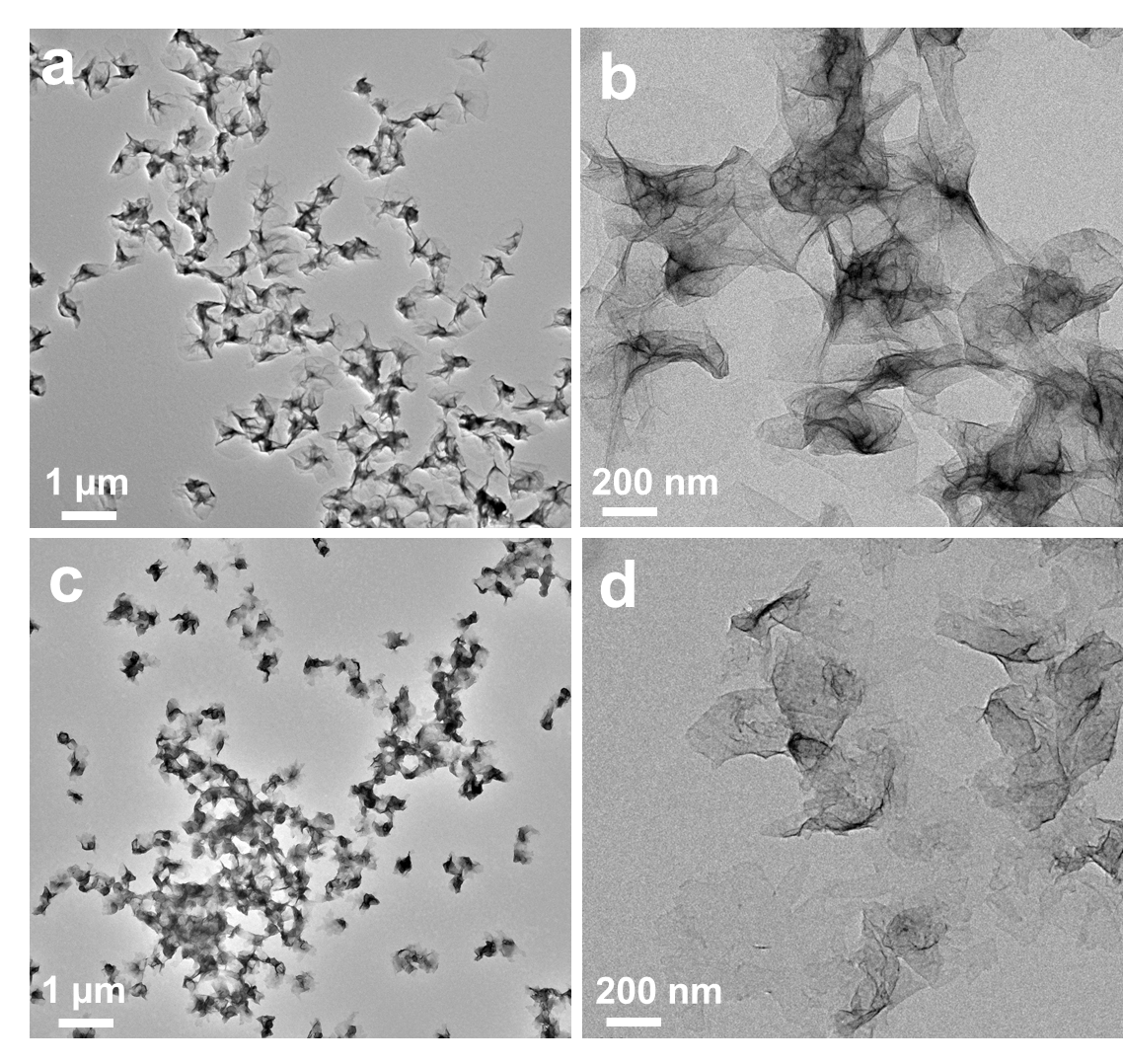
**

**Figure S1**. TEM image of (a,b) *a*-PdCu and (c,d) *c*-PdCu nanosheets.


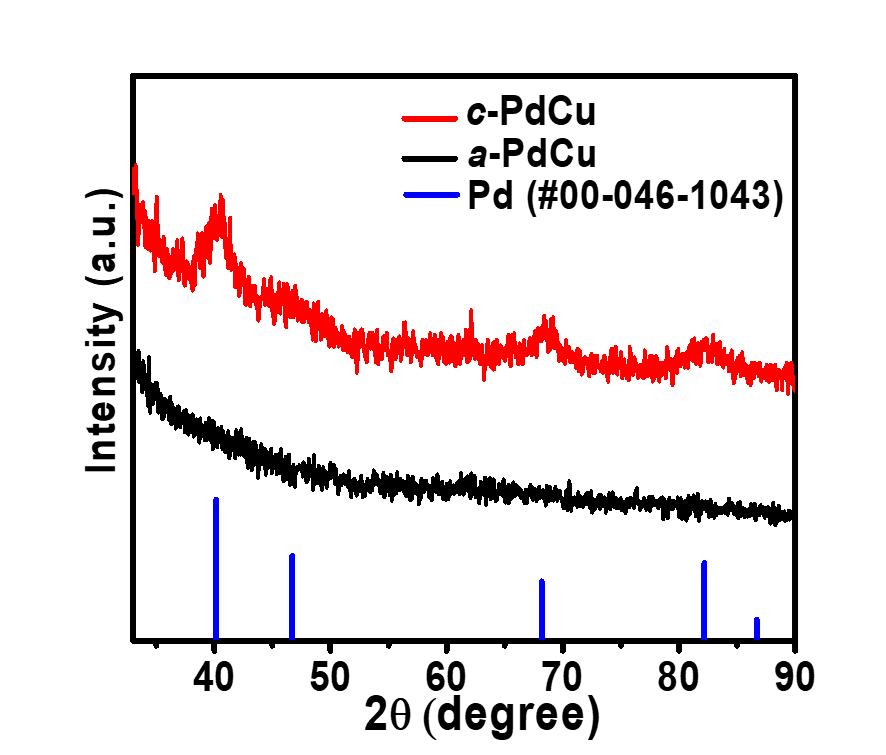


**Figure S2**. The XRD patterns of *a*-PdCu and *c*-PdCu. The standard XRD pattern of Pd (#00-046-1043) is used as reference.


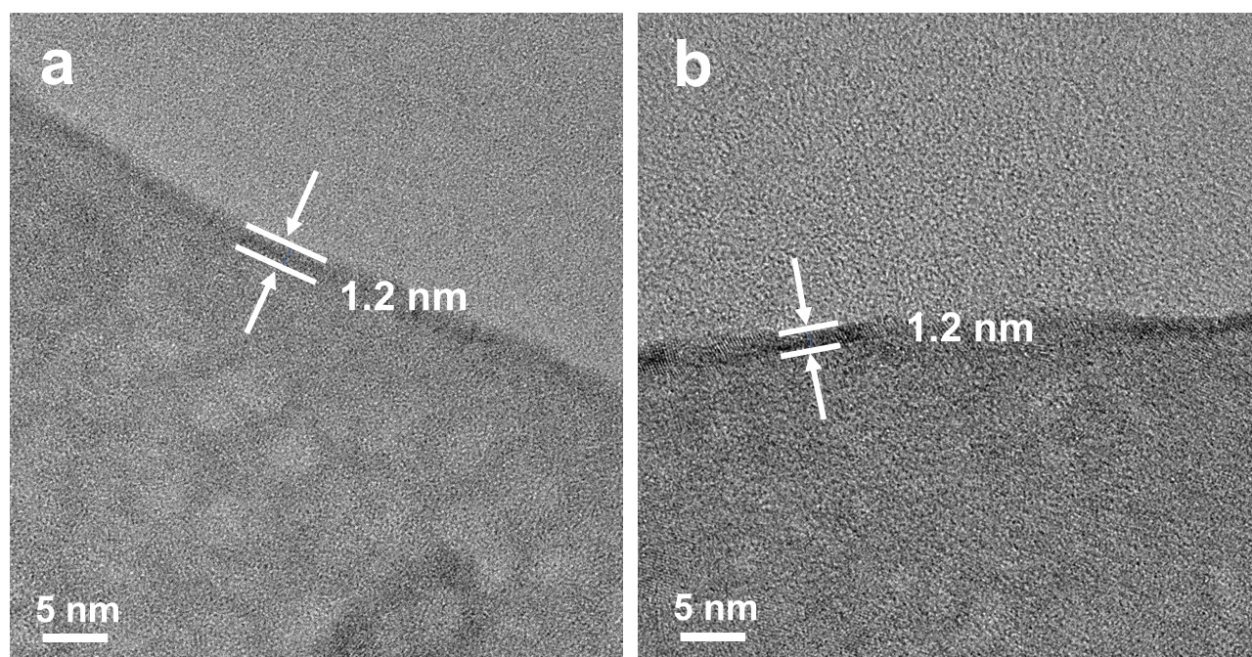


**Figure S3**. TEM images of a folded *a*-PdCu (a) and *c*-PdCu nanosheet (b), showing the folded edge with measured width of ~1.2 nm.


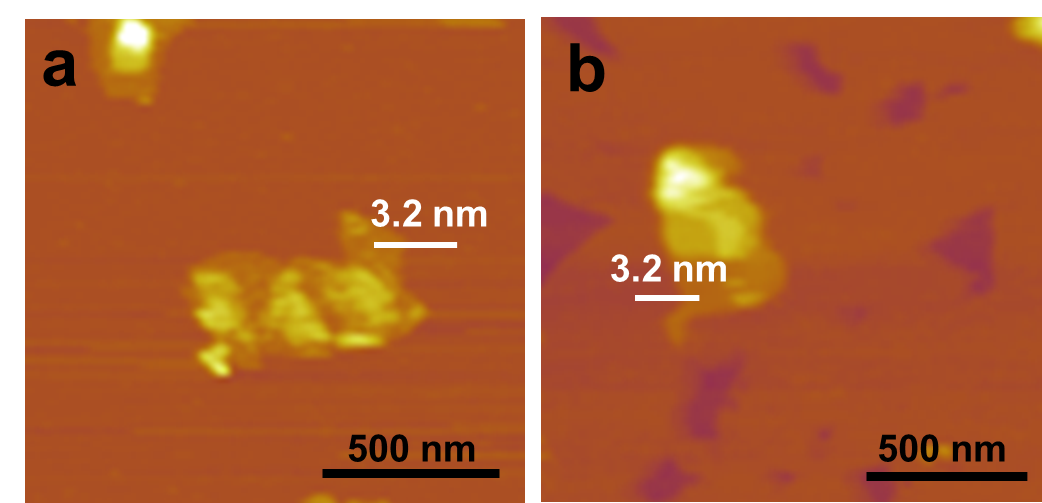


**Figure S4**. AFM images of (a) *a*-PdCu and (b) *c*-PdCu nanosheets, showing the thickness of ~3.2 nm.


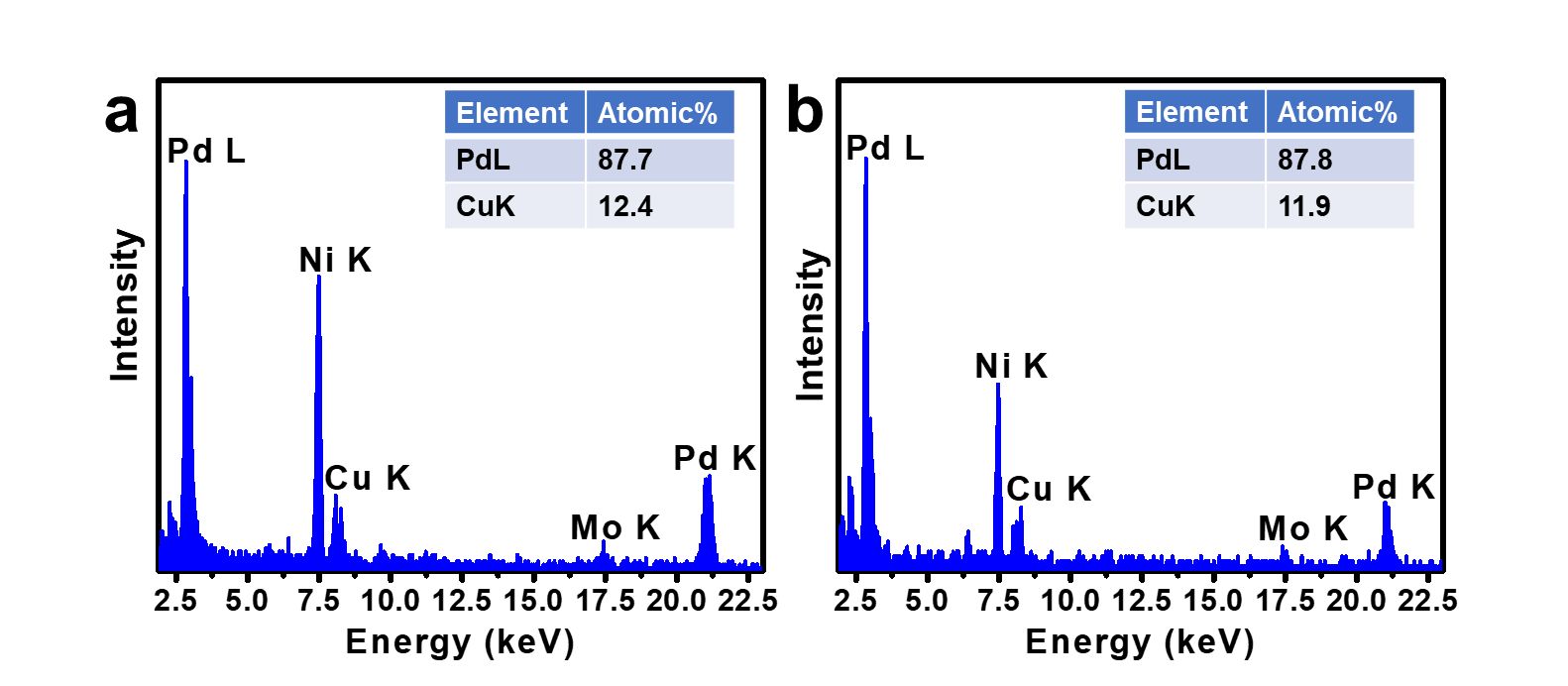


**Figure S5**. EDX spectra of (a) *a*-PdCu, and (b) *c*-PdCu nanosheets. The additional Ni K signal originates from the TEM nickel grid.


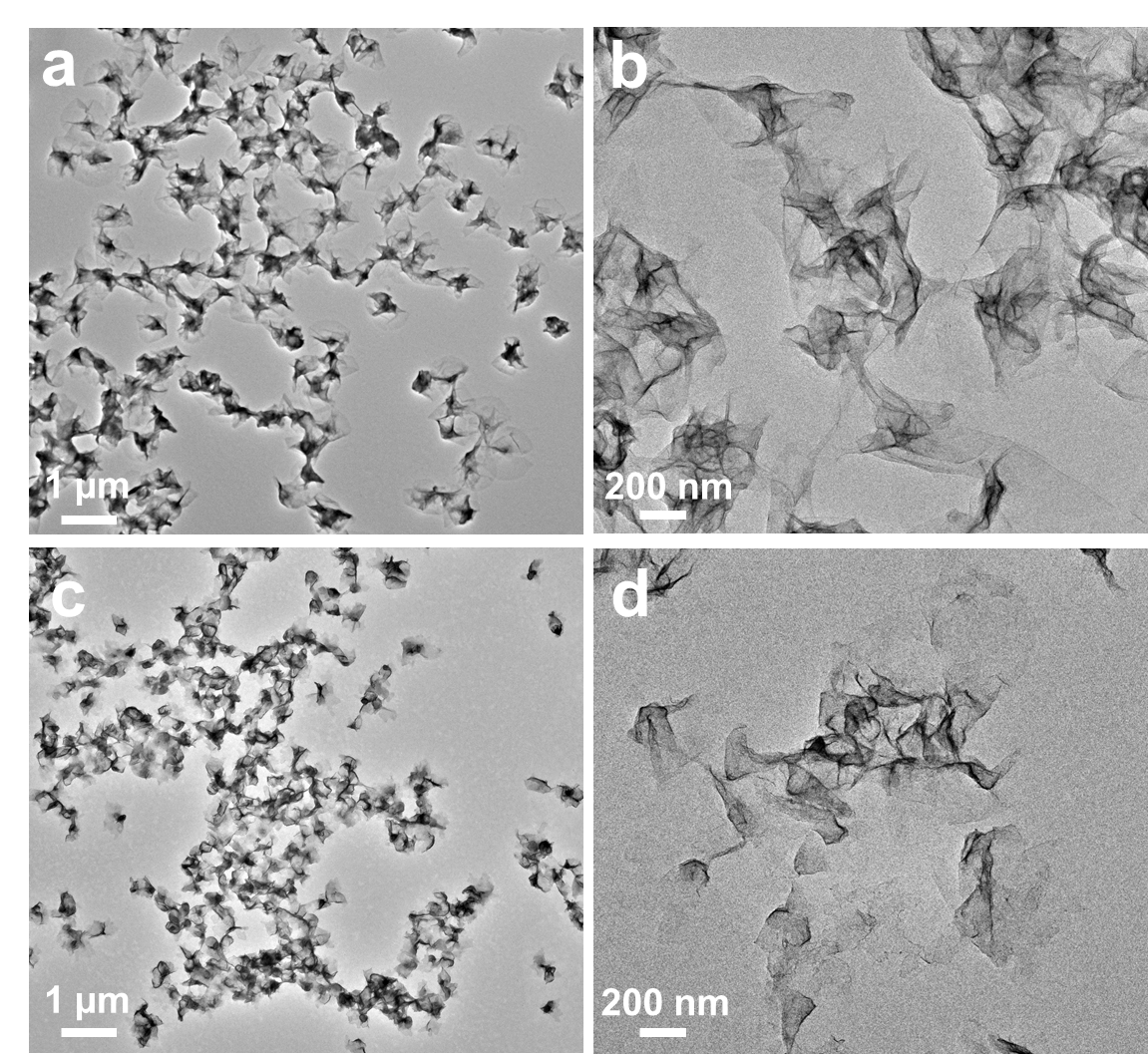


**Figure S6**. TEM images of (a,b) *a*-PdCu and (c,d) *c*-PdCu nanosheets after aging for 14 days.


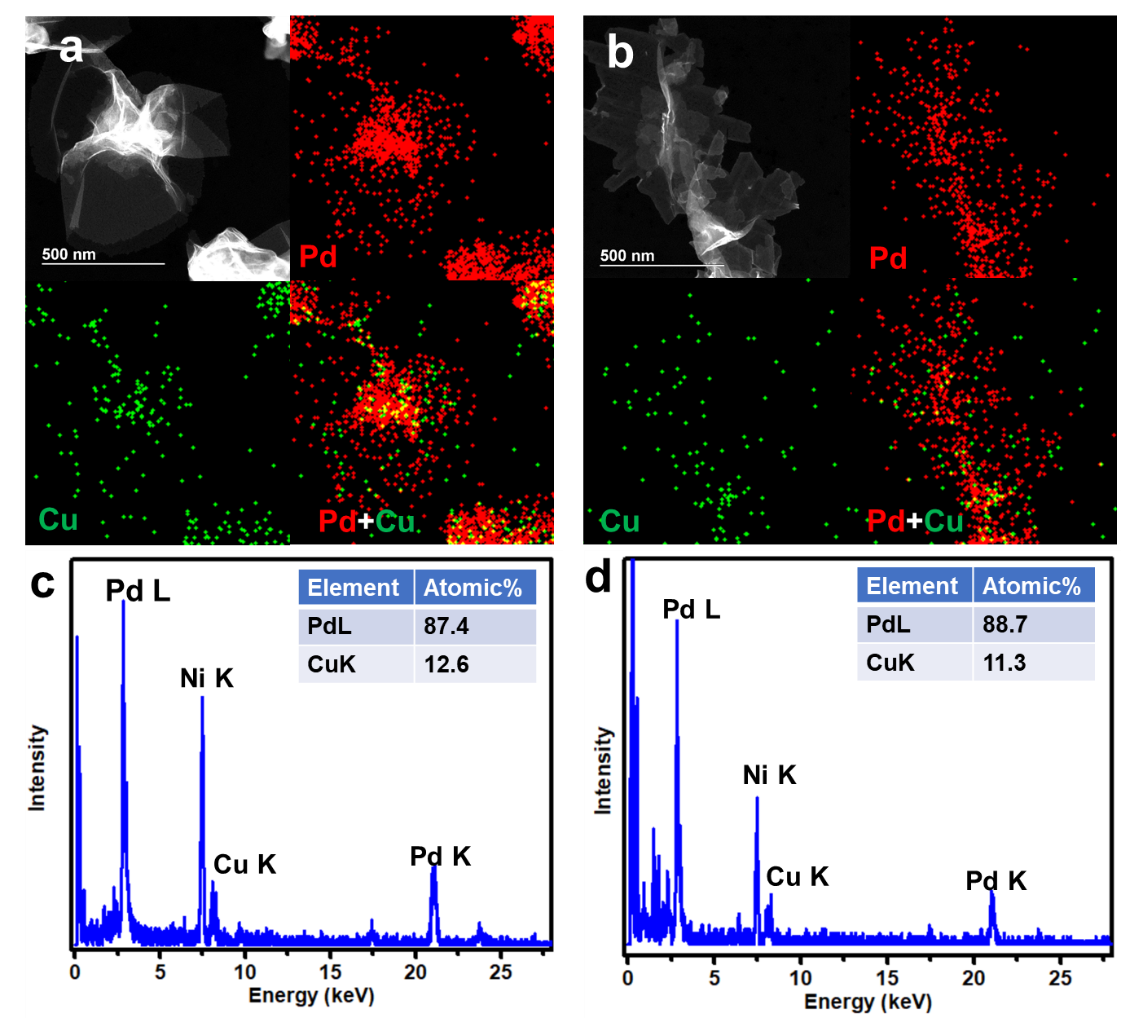


**Figure S7**. The STEM images and corresponding EDS mappings of (a) *a*-PdCu and (c) *c*-PdCu after aging for 14 days. The EDS elemental spectra of (c) *a*-PdCu and (d) *c*-PdCu after aging for 14 days.


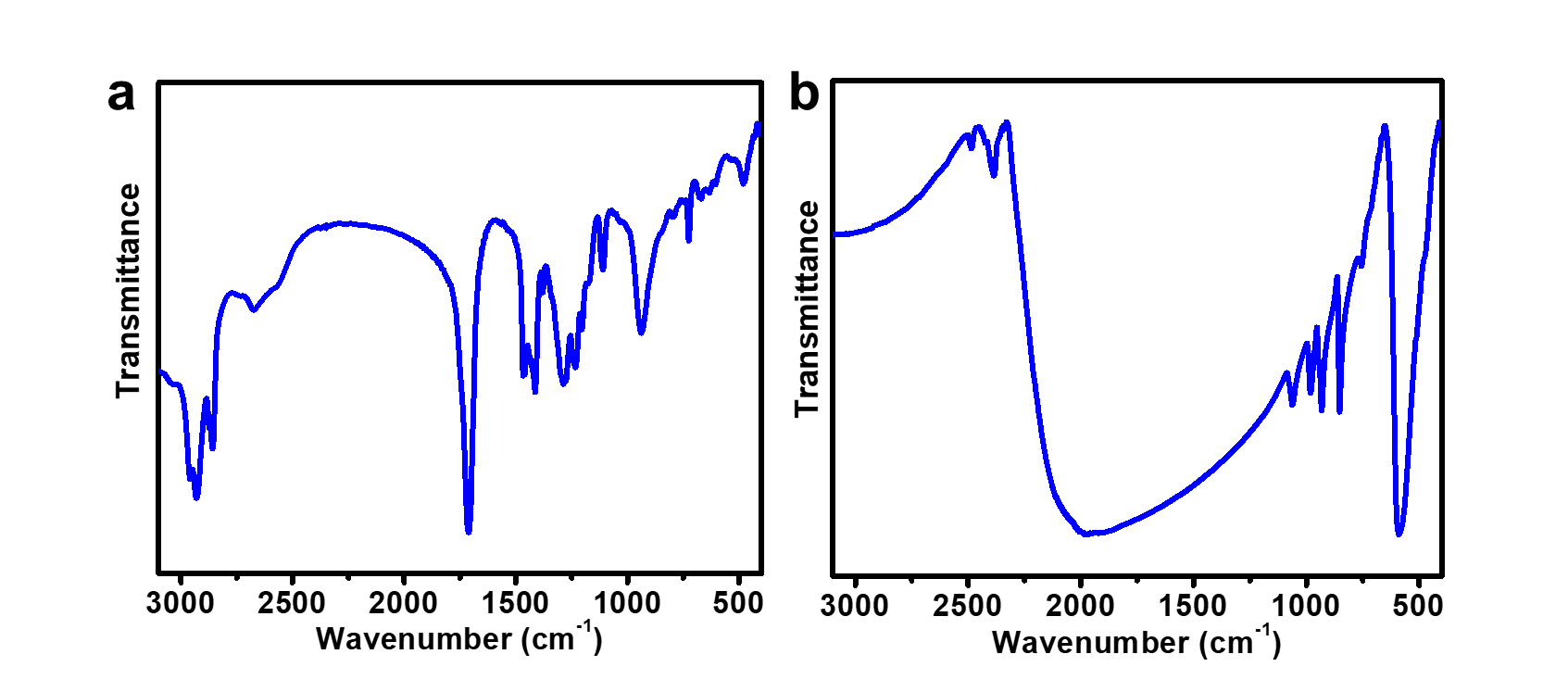


**Figure S8**. FTIR spectra of (a) octanoic acid and (b) Mo(CO)_6_.


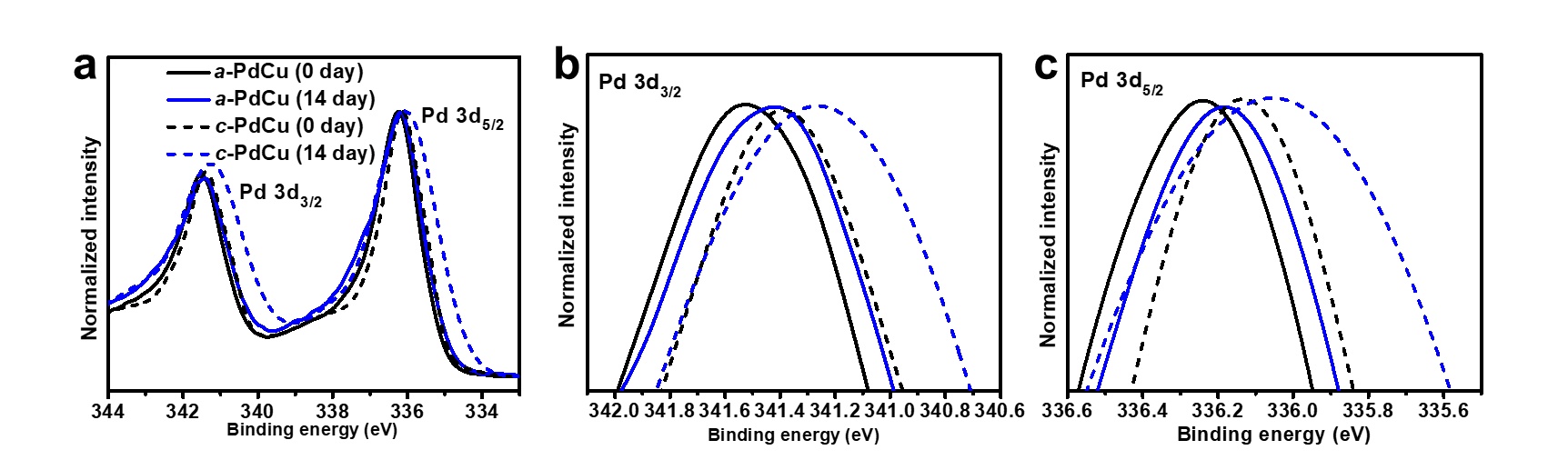


**Figure S9**. (a) Comparison of XPS spectra for the as-synthesized *a*-PdCu and *c*-PdCu (i.e. aging for 0 day), and the samples after aging for 14 days. The magnification of the normalized (b) Pd 3d_3/2_ and (c) Pd 3d_5/2_ peak.


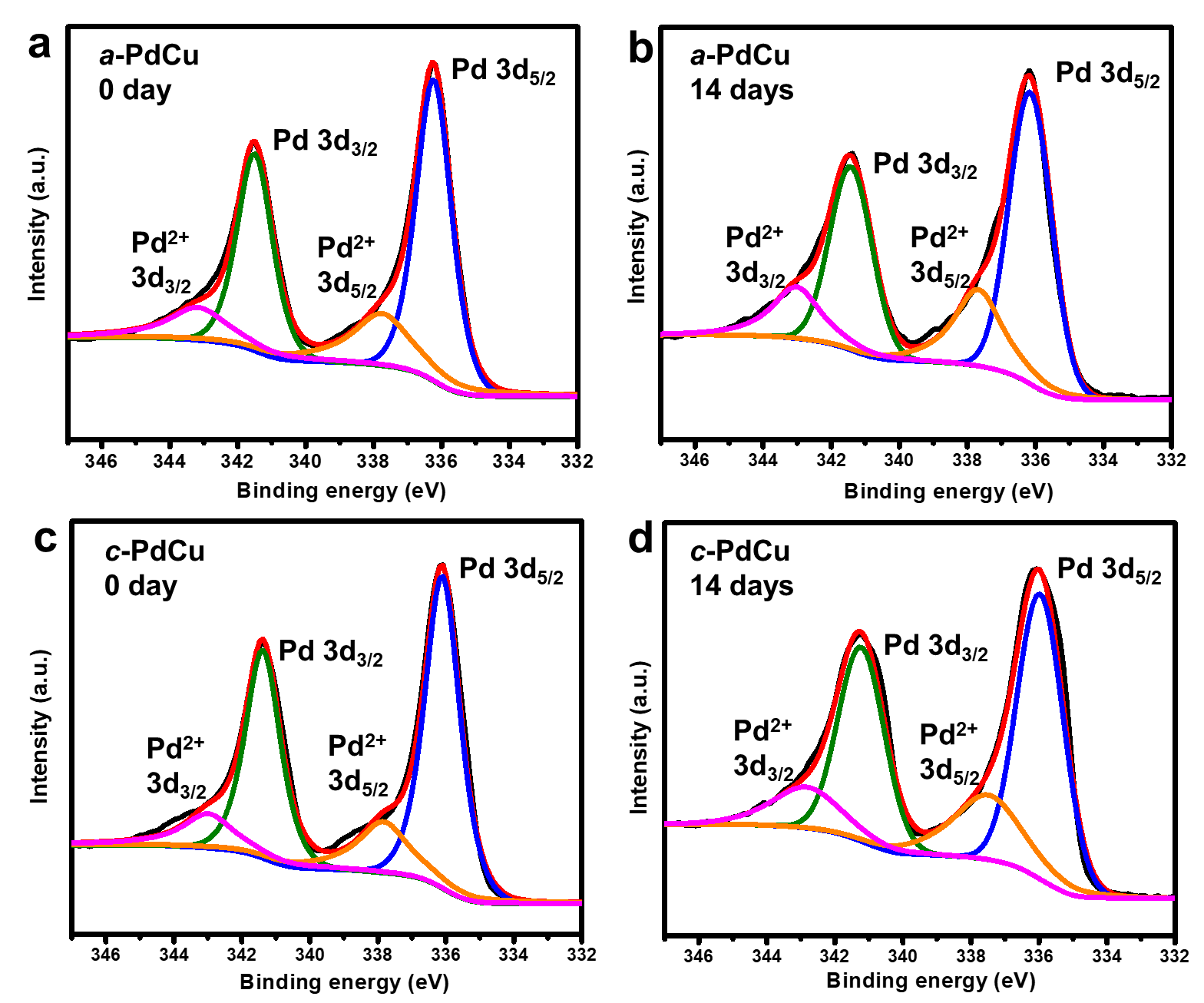


**Figure S10**. XPS spectra of Pd 3d : (a) the as-synthesized *a*-PdCu sample (i.e., aging for 0 day), (b) the *a*-PdCu sample after 14-day aging, (c) the as-synthesized *c*-PdCu sample (i.e., aging for 0 day), and (d) the *c*-PdCu sample after 14-day aging. The Pd^2+^/Pd^0^ ratios in the aforementioned four samples are estimated to be 0.29, 0.38, 0.30, 0.38, respectively, indicating that the Pd element in both *a*-PdCu and *c*-PdCu shows similar oxidation degree under our aging conditions.


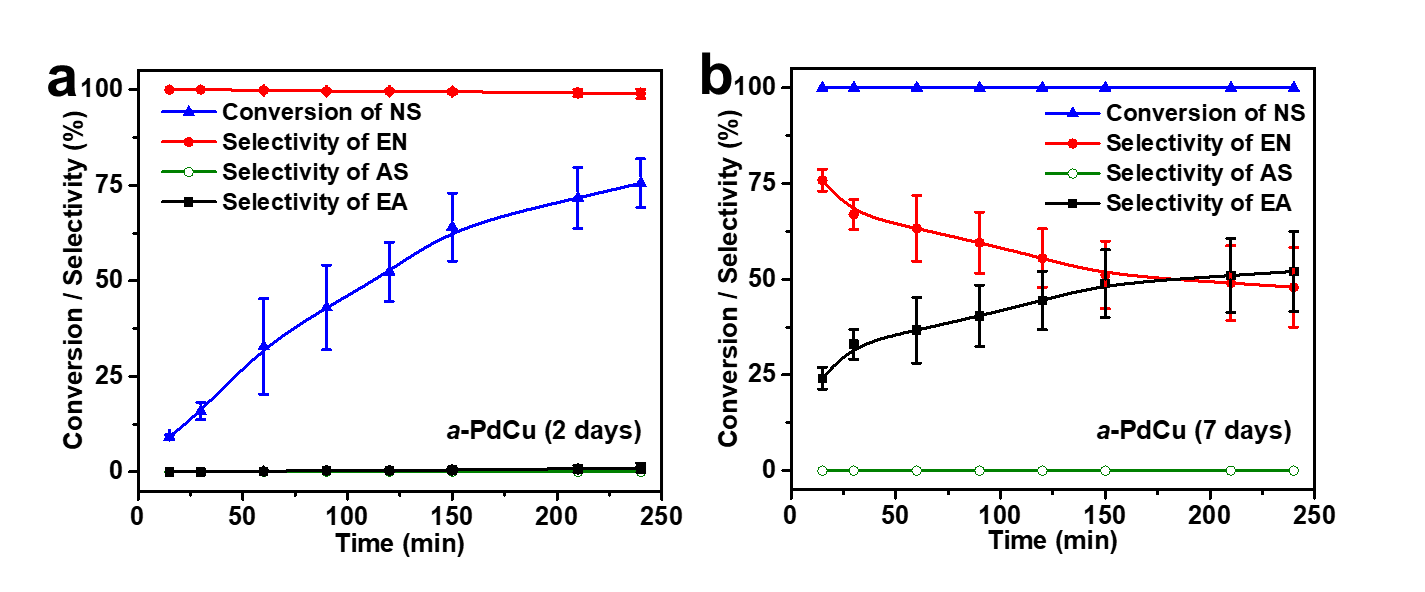


**Figure S11**. The kinetic curves showing the catalytic performance of *a*-PdCu after aging for (a) 2 days, and (b) 7 days.


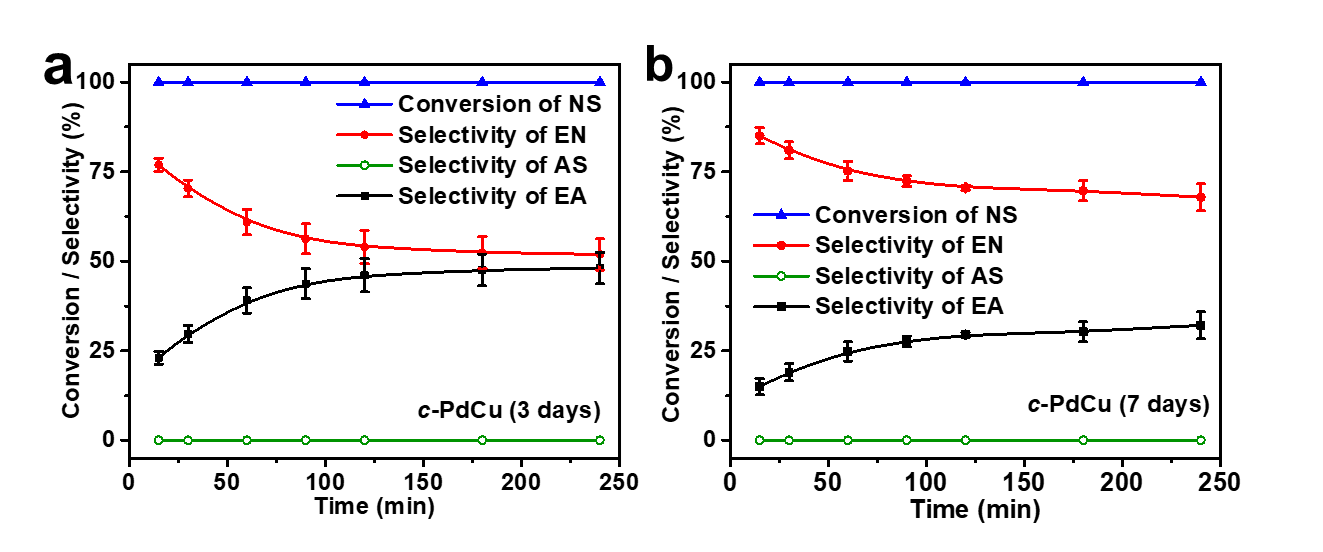


**Figure S12**. The kinetic curves showing the catalytic performance of *c*-PdCu after aging for (a) 3 days, and (b) 7 days.
